# Opa1-mediated mitochondrial dynamics is important for osteoclast differentiation

**DOI:** 10.17912/micropub.biology.000650

**Published:** 2022-10-15

**Authors:** Keizo Nishikawa, Hina Takegami, Hiromi Sesaki

**Affiliations:** 1 Laboratory of Cell Biology and Metabolic Biochemistry, Department of Medical Life Systems, Graduate School of Life and Medical Sciences, Doshisha University; 2 Department of Immunology and Cell Biology, WPI-Immunology Frontier Research Center, Osaka University; 3 Graduate School of Medicine/Frontier Biosciences, Osaka University; 4 Department of Cell Biology, Johns Hopkins University School of Medicine

## Abstract

Opatic atrophy 1 (Opa1) is a mitochondrial GTPase that regulates mitochondrial fusion and maintenance of cristae architecture. Osteoclasts are mitochondrial rich-cells. However, the role of Opa1 in osteoclasts remains unclear. Here, we demonstrate that *Opa1-* deficient osteoclast precursor cells do not undergo efficient osteoclast differentiation and exhibit abnormal cristae morphology. Thus, Opa1 is a key factor in osteoclast differentiation through regulation of mitochondrial dynamics.

**Figure 1. Expression of opatic atrophy 1 ( Opa1 ) and its knockout effect on osteoclastogenesis.**
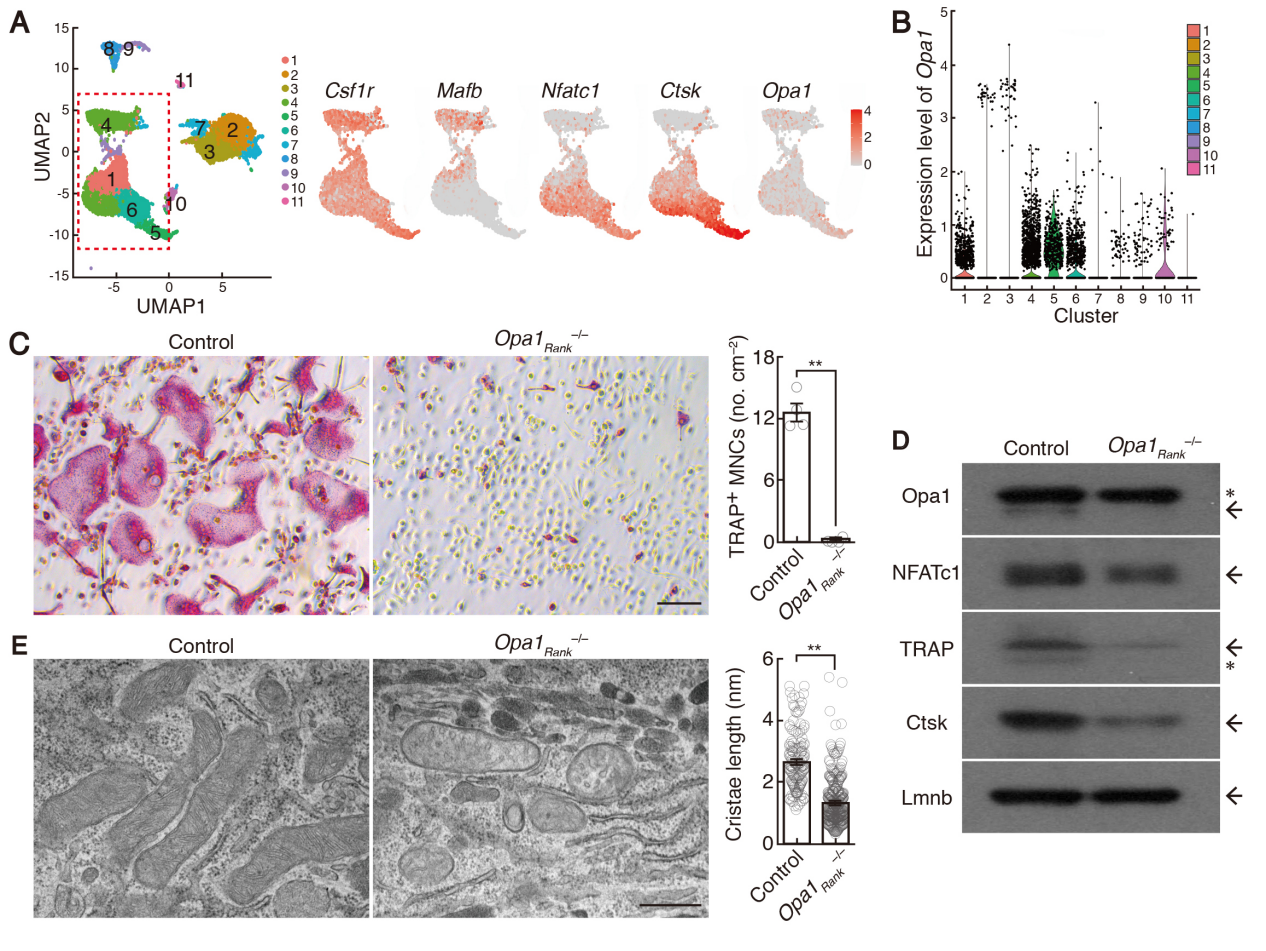
(A) Definition of the clusters present in the osteoclast culture system (left) and feature plots depicting single-cell gene expression of *Opa1* and canonical markers of osteoclast differentiation stages (right). According to the previous study (Tsukasaki *et al.* , 2020), cluster 4 and clusters 5 and 6 comprised of osteoclast precursor cells and mature osteoclasts, respectively (red dotted box). The pseudotime estimation showed that cluster 4 differentiated into clusters 5 and 6 by passing through cluster 1. (B) Violin plots showing the expression of *Opa1* in each cluster. (C) Effect of *Opa1* deficiency on osteoclastogenesis. TRAP-stained cells (left) and the number of TRAP-positive cells with more than three nuclei (right) are shown. Data from four independent experiments are shown as data points. Scale bar denotes 100 mm. (D) Protein expression of Opa1, NFATc1, TRAP, Ctsk and Lamin B (Lmnb) in control- and *Opa1 _Rank_^–/–^* -derived BMMs cultured in the presence of RANKL for 3 days. Asterisk denotes a nonspecific band. (E) Effect of *Opa1* deficiency on mitochondrial morphology. Transmission electron microscopy images (left) and the length of cristae (right). Data from 148 crista from 29 control mitochondria, and 332 crista from 82 *Opa1 _Rank_^–/–^* mitochondria from four independent experiments are shown as data points. Scale bar denotes 500 nm. Data are expressed as mean ± standard error of mean. ** *P* < 0.01 (t-test).

## Description

Mitochondria are critical for integrating several important metabolic processes involved in cell growth, survival, differentiation and cellular function (Kasahara & Scorrano, 2014; Mills *et al* , 2017; Vakifahmetoglu-Norberg *et al* , 2017). Mitochondria dynamically change their morphology by frequent fission and fusion (Friedman & Nunnari, 2014; Roy *et al* , 2015). Mitochondrial fission separates one into two, whereas fusion joins two mitochondria together. These processes are regulated by nuclear-encoded GTPases. Fusion is coordinated on the inner mitochondrial membrane by opatic atrophy 1 (Opa1) and on the outer mitochondrial membrane (OMM) by mitofusin 1 and 2 (Mfn1 and Mfn2). Fission is controlled by dynamin-related protein 1 (Drp1), whose mitochondrial recruitment is mediated by multiple OMM-bound proteins, such as Fis1, Mff, Mid49, and Mid51 (Bui & Shaw, 2013; MacVicar & Langer, 2016; Tamura *et al* , 2011). Osteoclasts are specialized multinucleated giant cells involved in bone homeostasis through bone resorption (Takayanagi, 2007). Mitochondrial biogenesis is promoted during osteoclast differentiation; therefore, mature osteoclasts contain abundant mitochondria, which are rich in crista (Ishii *et al* , 2009; Lemma *et al* , 2016). Owing to the fact that Drp1 is required for osteoclast differentiation (Jeong *et al* , 2021), mitochondrial division is considered an essential process for osteoclastogenesis. Considering that mitochondria are increased in size in mature osteoclasts, mitochondrial fusion should be enhanced. However, the role of Opa1 in osteoclastogenesis remains unclear.

Osteoclast differentiation was evaluated *in vitro* by counting multinucleated cells (MNCs) positive for the osteoclast marker, tartrate-resistant acid phosphatase (TRAP), following stimulation of bone marrow-derived monocyte/macrophage precursor cells (BMMs) with receptor activator of nuclear factor-κB ligand (RANKL), and in the presence of macrophage colony-stimulating factor (M-CSF)(Nishikawa *et al* , 2010a; Nishikawa *et al* , 2021) *.* To assess *Opa1* expression during osteoclast differentiation, we examined a previously published single-cell RNA sequencing (scRNA-seq) dataset of *in vitro* osteoclast differentiation (Tsukasaki *et al* , 2020). After reprocessing, quality control, and normalization, the data showed that *Opa1* is constantly expressed during osteoclast differentiation (Figure 1, A and B). Next, to investigate the role of Opa1 in osteoclast differentiation, we crossed *Opa1 ^flox/flox^* mice (Zhang *et al* , 2011) with *Rank* ^Cre/+^ mice (Maeda *et al* , 2012) to disrupt the *Opa1* gene in the osteoclast lineage ( *Opa1 _Rank_^–/–^* ). This was followed by an *in vitro* osteoclast differentiation assay using *Opa1 _Rank_^–/–^* BMMs. RANKL-induced formation of TRAP-positive MNCs in *Opa1 _Rank_^–/–^* BMMs was lower than that in the control BMMs (Figure 1C). To validate the impairment of osteoclast formation by Opa1 loss, we measured the expression levels of Opa1 and several osteoclast-specific markers, such as nuclear factor of activated T cells 1 (NFATc1), cathepsin K (Ctsk) and TRAP. We observed downregulation in the expression of these proteins in *Opa1 _Rank_^–/–^* BMMs stimulated with RANKL for 2 days (Figure 1D). These results suggest that Opa1-mediated mitochondrial fusion is required for osteoclastogenesis. Considering that Opa1 is a key regulator of cristae remodeling and is involved in shaping cristae (Buck *et al* , 2016; Cogliati *et al* , 2013; Panek *et al* , 2020), we investigated the effect of *Opa1* knockout on cristae morphology. Transmission electron microscopy revealed that the cristae of control mitochondria were flat structures while *Opa1 _Rank_^–/–^* mitochondria exhibited disorganized cristae morphology with vesicular cristae structure (Figure 1E). These finding suggests that Opa1-mediated cristae remodeling is required for osteoclastogenesis.

## Methods

**Mice and bone analysis**

We generated and genotyped *Opa1 ^flox/flox^* and *RANK* ^Cre/+^ mice as previously described (Maeda *et al.* , 2012; Zhang *et al.* , 2011). *Opa1 ^+/+^* , *Opa1 ^flox/+^ and Opa1 ^flox/flox^* littermate mice that did not carry the Cre recombinase were used as controls. Following their birth, all mice were maintained under specific pathogen-free conditions. All animal experiments were approved by the Institutional Animal Care and Use Committee of both Doshisha University and Osaka University. All the strains featured a C57BL/6 background. Two-week-old sex-matched mice were used in the experiments. Animals were randomly included in the experiments based on the genotyping results.

**Cell culture**

*In vitro* osteoclast differentiation was performed as previously described (Iwamoto *et al* , 2016; Nishikawa *et al* , 2013; Nishikawa *et al* , 2015). Briefly, bone marrow-derived cells cultured with 10 ng/ml M-CSF (Miltenyi Biotec) for 2 days were used as osteoclast precursor cells and BMMs, and were further cultured with 50 ng/ml RANKL (PeproTech) in the presence of 10 ng/ml M-CSF for 3 days. TRAP-positive MNCs (TRAP+ MNCs) having more than three nuclei were counted.

**Transmission electron microscopy**

BMMs cultured on Cell Desk polystyrene cover slip (Sumitomo Bakelite Co., Ltd., Japan) were fixed for 24 hrs at 4°C in 2% formaldehyde and 2.5% glutaraldehyde in 0.1M cacodylate buffer (pH7.4) containing 0.01% calcium chloride. Each sample was washed for 5 min in 0.1M cacodylate buffer (pH7.4) containing 7% sucrose for three times. Cells were post-fixed for 1h with 1% osmium tetroxide and 0.5% potassium ferrocyanide in 0.1M cacodylate buffer (pH7.4), dehydrated in a graded ethanol series, and embedded in Epon812 (TAAB Co. Ltd., UK). Ultrathin sections (80 nm) were stained with saturated uranyl acetate and lead citrate solutions. Electron micrographs were obtained using a JEM-1400 plus electron microscope (JEOL, JP) at 80 kV.

**Immunoblot analysis**

Immunoblot analysis was performed as described previously (Nishikawa *et al* , 2010b). Briefly, the cell lysates were subjected to immunoblot analysis using antibodies specific for Opa1 (Abcam, ab157457), NFATc1 (Santa Cruz Biotechnology, sc-7294), TRAP (Santa Cruz Biotechnology, sc-30833), Ctsk (Daiichi Finechemical, F-95) and Lmnb (Santa Cruz Biotechnology, sc-6217). Whole-cell extracts were prepared by lysis in a radioimmunoprecipitation assay buffer.

**Single-cell RNA-sequencing analysis**

Gene expression data of scRNA-seq (GSE147174) obtained from NCBI’s Gene Expression Omnibus ( https://www.ncbi.nlm.nih.gov/geo/ ) were processed and analyzed using the Seurat R package (v.4.0.6) as described previously(Tsukasaki *et al.* , 2020). Briefly, cells expressing less than 200 genes and more than 5% ofmitochondrial geneswere defined as poor-quality data and excluded. After normalization and scaling, the top 2,000 variable genes were selected by directly modelling the mean-variance relationship inherent in single-cell data. We performed dimensionality reduction using principal-component analysis (PCA) and visualized single cells on a uniform manifold approximation and projection (UMAP) plot according to gene expression.
